# Investigating factors affecting on medical sciences students’ intention to adopt mobile learning

**DOI:** 10.1186/s12909-019-1831-4

**Published:** 2019-10-21

**Authors:** Seyyed Mohsen Azizi, Alireza Khatony

**Affiliations:** 1 Clinical Research Development Center of Imam Reza Hospital, Kermanshah University of Medical Sciences, Kermanshah, Iran; 2 Health Institute, Social Development and Health Promotion Research Center, Kermanshah University of Medical Sciences, Kermanshah, Iran

**Keywords:** Mobile learning, Adoption, Readiness, Medical education, Student, Theory of planned behavior

## Abstract

**Background:**

Mobile learning (m-learning) provides a good opportunity for students’ lifelong learning. The design and implementation of effective and successful mobile learning requires identification of factors that affect m-learning. The aim of this study was to investigate the factors that affect the intention of students of medical sciences to adopt mobile learning based on theory of planned behavior (TPB).

**Methods:**

In this cross-sectional study, 332 students of medical sciences were randomly selected. The study tool was a based a questionnaire that had been designed based on TPB model. Descriptive statistics (mean, standard deviation, frequency and percentage) were calculated. In order to determine the standardized factor loading and assess the study hypotheses, structural equation modeling was used. Composite reliability, average variance extracted, and standardized factor loading were used to determine the convergent validity.

**Results:**

The mean of mobile learning readiness was 3.59 ± 0.83. Among the TPB structures, the structures of attitude (β = 0.525) and behavioral control (β = 0.318) had positive and significant effect on the intention to adopt m-learning (*P* ≤ 0.01). However, the structure of subject norm did not have a significant effect on the intention to adopt m-learning. In general, attitude, behavioral control and subject norm structures were 0.675 determinants of the intention to adopt m-learning (r^2^ = 0.675).

**Conclusions:**

In this study Mobile learning readiness of the students was at moderate level. Also the results indicated Positive and significant effect of attitude and behavioral control on the intention of students to accept m-learning. The TPB-based model was a suitable model for identifying psychological factors that affect the intention of students of medical sciences to adopt m-leaning. In order to increase the students’ acceptance of mobile learning, we suggest that, other psychological, behavioral, social, and cultural factors that affect the acceptance of m-learning should be identified. Educational programs are also suggested to be introduced to students to familiarize them with the m-learning and its application in learning process.

## Background

In recent years, researchers have focused on the various aspects of modern technologies in teaching-learning process [1]. Mobile learning, as one of the emerging forms of e-learning, is one of the concepts that have attracted the attention of researchers in the field of teaching-learning [2–5]. Mobile learning involves the use of mobile technology, alone or in combination with various forms of information and communication technology [6]. Mobile learning (ML) creates many opportunities and challenges including; increasing access to educational opportunities, facilitating personal learning, providing immediate feedback, learning at any time and place, making a bridge between formal and informal learning, reducing barriers to education in remote areas, helping learners with disabilities, improving communication, and being cost-effectiveness [6]. However, ML also has some challenges. One of these challenges is the pedagogical challenge, such as the design of educational content compatible with mobile learning. Cultural and social challenge is another challenge of ML. Cultural norms and social concerns about the acceptance of mobile learning, the learner-centered environment of learning and the acceptance of mobile as an educational tool by educators and students are among the cultural-social challenges of ML [7]. Also, another challenge and limitation of ML is the technological challenge. The small size of mobile phone’s LCD screen, the high cost of smartphone, memory limitation and the short battery lifespan of the mobile phones are among the most important technological challenges [8]. Despite the aforementioned challenges and limitations, the use of smartphone as a tool for learning in universities has been widely considered [9]. In the field of medical education, the tendency to use mobile technology for learning is on the rise day-by-day [10]. Results of a systematic review indicated that, the use of mobile technology in medical sciences is increasing [11]. Another systematic review showed that, the use of mobile technology in medical education increases students’ knowledge and clinical skills [12].

In this regard, empirical evidence in the field of medical education suggests that learners who use mobile apps for learning have better performance compared to learners who only use the traditional method [13]. Evidence also suggests that ML applications have a positive and significant impact on students’ learning [14, 15].

The design and implementation of m-learning requires its acceptance by the target community. Various factors affecting the adoption/acceptance of mobile learning have been investigated in many studies [4, 16, 17]. One of the theoretical frameworks is the Theory of Planned Behavior (TPB) which is used to identify the factors that influence the adoption of mobile learning. This theory was presented by Ajzen (1991) In this theory, the three structures of Attitude, Subject Norm and Perceived Behavior Control are used to predict the learning intention [18]. The Attitude represents the positive or negative beliefs and judgments of individual about the consequences and behavioral characteristics. The Subject Norm refers to an individual’s perception of social pressures of important people (such as family, teachers, and classmates) in whether to act or not. The Perceived Behavior Control indicates the likelihood of success (difficult or easy) of a behavioral effort, and also indicates how much people have control over a behavior [19, 20]. Based on this theory, human behavior is influenced by behavioral beliefs, normative beliefs and control beliefs [21].

In some studies, TPB and other technology adoption models and techniques have been used to examine the factors affecting learning acceptance through mobile or e-learning. In this regard, Reza et al. (2017) showed that TPB variables including attitudinal, normative, and control beliefs have a positive and significant effect on the acceptance of mobile learning in Pakistani students [22]. Results of a study by Cheon et al. (2012) also revealed that TPB is a determinant of intention of American students to use m-learning [17]. Dai (2015) showed that ATT, SN, and BC have a significant effect on the intention of Chinese students to accept m-learning [23].

Briz-Ponce et al. conducted a study on learning through mobile technology in Portuguese students. The framework of this study was based on TAM and UTAUT. Their results showed that, the factor of “social impact” had a positive effect on the attitude and behavioral intention in using m-learning [5]. The results of a study conducted in Brazilian universities showed that attitude and subjective norm had a positive and significant effect on the intention to use e-learning [24]. Findings of Yeap et al. also showed the positive effect of attitude, subjective norm, and behavioral control structures on the intention of Malaysian students to use m-learning [20]. In general, identification of factors that affect students’ intention to accept m-learning plays a crucial role in the design and implementation of effective m-learning system. Therefore, in this study, we intended to investigate the factors affecting the intention of Iranian students of medical sciences to adopt m-learning using TPB-based model.

## Methods

### ConÎptual model and hypothesis

**Fig. 1:**
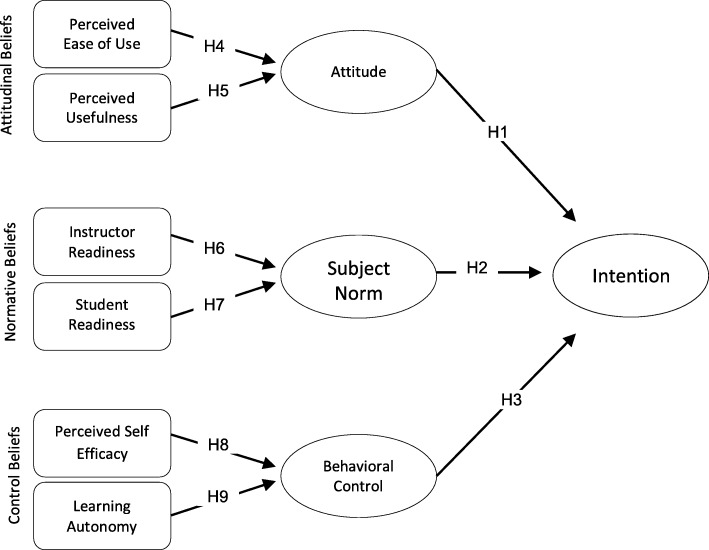
Conceptual model based on the theory of behavioral planned

The conceptual model consists of 10 structures that are designed based on TPB. The main structures include “attitudinal structures”, which contain Attitude, Subject Norm, and Behavioral Control. Based on the conceptual Model, the “External Beliefs” include attitudinal beliefs, normative beliefs, and control beliefs. External beliefs also include Perceived Ease of Use and Perceived Usefulness. Normative beliefs include Instructor Readiness and Student Readiness. A control belief includes Perceived Self-efficacy and Learning Autonomy. Behavioral intention includes the intention structure. The hypotheses of the study and the direction of each hypothesis are plotted in the model. It should be noted that, in the discussion section, we have explained the perimeter of each of the structures according to the findings (Fig. 1).

H1: The students’ attitude towards m-learning significantly influences their intention to adopt m-learning.

H2: The students’ subjective norm towards m-learning significantly influences their intention to adopt m-learning.

H3: The students’ perceived behavioral control towards m-learning significantly influences their intention to adopt m-learning.

H4: The students’ perceived ease of use towards m-learning significantly influences their attitude to adopt m-learning.

H5: The students’ perceived usefulness towards m-learning significantly influences their attitude to adopt m-learning.

H6: The instructor’s readiness towards m-learning significantly influences on subjective norm for m-learning.

H7: The student’s readiness towards m-learning significantly influences on subjective norm for m-learning.

H8: The students’ perceived self-efficacy towards m-learning significantly influences their behavioral control with m-learning.

H9: The students’ perceived learning autonomy towards m-learning significantly influences their behavioral control with m-learning.

### Study design

This cross-sectional and descriptive-analytical study was conducted from March to July 2018 at the schools of KUMS.

### Sample and sampling method

The study population consisted of students in the second semester of 2017–2018 academic year. Correction sample size was used to calculate the sample size. Considering the correlation coefficient of (*r* = 0.88) of the structures of ML in the study of Cheon et al. (2012), the confidence level of 0.95 and the test power of 0.90, the sample size was calculated to be 9 people (*n* = 9) [17]. However, in the present study the sample size was determined to be 357 people using a Cochrane formula. The samples were selected by stratified random sampling method. The KUMS faculties, including the faculties of Medicine, Paramedicine, Dentistry, Pharmacy, Nursing & Midwifery, and Health, formed the sampling classes. Sampling was done in each class by stratified random method using a random table of numbers. According to the number of students in each faculty, a percentage of them were selected to represent the whole university. For this purpose, the list of students at each faculty was obtained and numbered. Then, using a random table of numbers, 357 students were selected from different faculties and programs (*n* = 332, 92% response rate). The inclusion criteria were; studying at the second semester of the academic year 2017–2018, willing to participate in the study and being at the term 2 or above. The incomplete filling of the questionnaires was considered as an exclusion criterion.

### Instrument

The data gathering tool was a two-part questionnaire. The first part was dedicated to personal information, including sex, age, education level, and educational faculty, smartphone or tablet ownership, and the duration of mobile use per year. The second part included the Theory of planned behavior (TPB)-based questionnaire. This questionnaire was prepared by Cheon et al., [17]. Validity and reliability of this questionnaire have been verified by Yeap et al. [20]. Cheon et al. have also verified the validity of this tool by a convergent and divergent method, and its reliability coefficient has been reported to be over 0.70 for all structures using Cronbach’s Alpha [17]. In this study, content validity method was used to determine the validity of questionnaire. Since this tool has not been used in Iran yet, a translator specializing in English and medical education translated the questionnaire into Persian at first, and then the second native translator, converted the translation into the original version of the questionnaire. After reviewing and fixing the problems, the final translation was agreed upon. Then, a reverse translation was performed and desired matching was ensured for all items of the questionnaires.

Validity of the questionnaire was assessed by content validity method, which included two qualitative and quantitative methods. In the qualitative section, the questionnaire was given to 12 faculty members specializing in medical education and educational psychology. They were asked to express their views regarding the questions of the questionnaire, and based on their comments, the necessary changes were made. In the quantitative section, the CVR and CVI indexes were calculated to be 0.83 and 0.89, respectively based on the expert opinions, which indicated the acceptable content validity of the questionnaire. In order to measure the reliability of the tool, internal consistency was used, and the questionnaire was distributed among 30 students, which resulted in the Cronbach’s Alpha of 0.92. It should be noted that, these students did not enter the main study.

The ML questionnaire consisted of 30 items and 10 structures. These structures included; perceived ease of use (PEU), perceived usefulness (PU), attitude, subjective norm (SN), perceived behavioral control (PBC), instructor’s readiness (IR), student’s readiness (SR), perceived self-efficacy (PSE), learning autonomy (LA), and intention.

PEU and PU structures are related to attitudinal beliefs. These structures are based on the technology of acceptance model [25]. The IR and SR structures are related to normative beliefs. Since the students and faculty members are among the most important groups in higher education, the readiness of instructors and students was chosen as two main structures of normative beliefs [17]. The PSE and LA structures are related to control beliefs. Self-efficacy refers to an individual’s assessment of his/her abilities and skills [26]. Autonomy also expresses the individual’s ability to control his/her learning process [17]. Therefore, due to the importance of learner’s role in the m-learning process, two structures PSE and LA were selected. Each of these structures had 3 items on a 5-point Likert scale, including strongly disagree, disagree, neutral, agree, strongly agree, which were scored from 1 to 5, respectively.

**Fig. 2:**
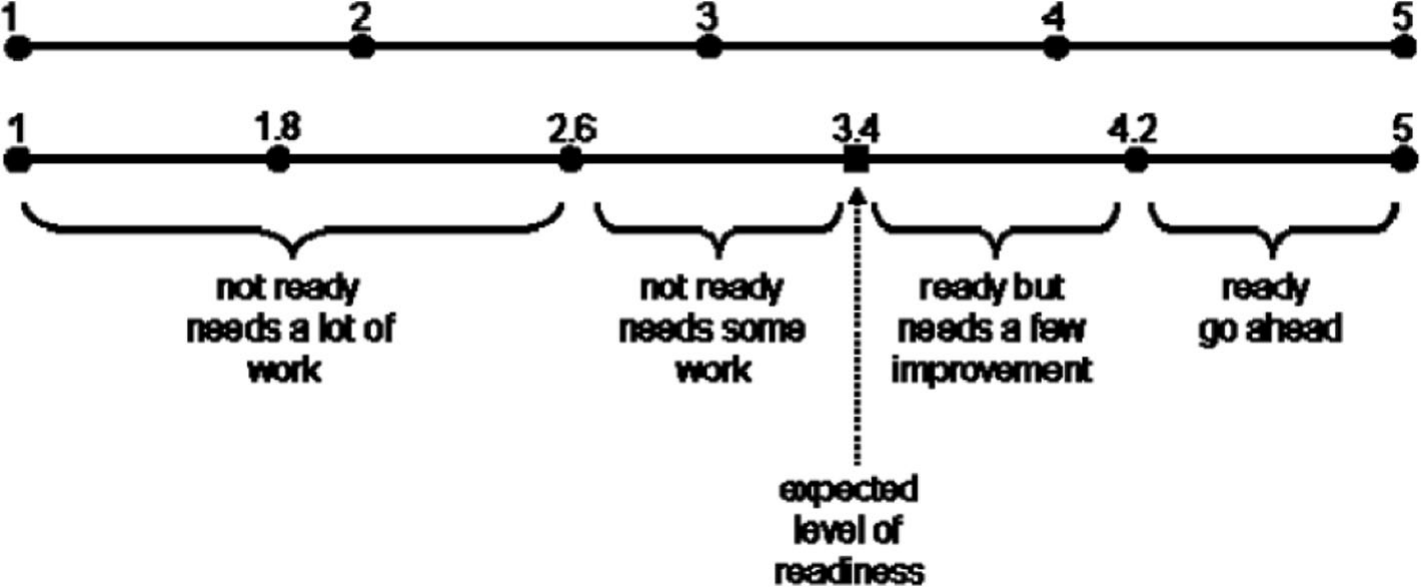
Assessment model of the Mobile Learning Readiness

To determine the level of students’ MLR, the Aydin and Tasci (2005) study method was used [27]. According to their study, the average score of readiness, or the boundary between readiness and non-readiness for ML, was determined at 3.4, and based on the acquired score, the samples were classified in one of the following categories; not ready and needs a lot of work (1–2.6), not ready and needs some work (2.6–3.4), ready but needs a few improvement (3.4–4.2), and ready to go ahead (4.2–5) (Fig. 2).

### Data gathering

In order to conduct the study, the necessary permission was obtained from the University’s Deputy for Research & Technology. The researcher then attended the KUMS’ faculties and obtained the names of students at each faculty from the education department. The students’ list was numbered and then samples were selected according to the random table of numbers. The researcher referred to them according to the students’ classroom program. First, the objectives of the study were explained to the students and those, who were willing to participate in the study, entered the study. Then the questionnaire was given to them and collected from them after completion. Also, if any person did not want to participate in the study, he/she was replaced by the person above or below him/her on the students’ list.

### Data analysis

Data were analyzed using the 22th version of Statistical Package for Social Sciences (SPSS v.22.0; SPSS Inc., Chicago, IL, USA) and 23th version Amos.

The SPSS software version 22 was used for descriptive statistics (frequency and percentage) and to determine the reliability of findings. The Cronbach’s alpha index was used to check the reliability of latent variables. Acceptable score for this index was equal to or higher than 0.7 (a ≥ 0.7). In order to determine the standardized factor loading and assess the study hypotheses, structural equation modeling was used in Amos-23 software. Composite reliability, average variance extracted, and standardized factor loading were used to determine the convergent validity. The following formulas were used to calculate CR and AVE [31, 32]:

$$ \mathrm{CR}=\left(\Sigma\ \mathrm{standardized}\ \mathrm{factor}\ \mathrm{loading}\right)\ 2/\left(\left(\Sigma\ \mathrm{standardized}\ \mathrm{factor}\ \mathrm{loading}\right)\ 2+\left(\mathrm{measurement}\ \mathrm{error}\ \mathrm{of}\ \mathrm{the}\ \mathrm{measured}\ \mathrm{variable}\right)\right) $$

$$ \mathrm{AVE}=\left(\Sigma\ \mathrm{standardized}\ \mathrm{factor}\ \mathrm{loading}\right)\ 2/\mathrm{N} $$

Acceptable score for factor loading and CR were set at equal to or higher than 0.7. For AVE, the acceptable score was equal to or higher than 0.5. Also, to investigate the discriminant validity, the square root of AVE in each structure was compared with the correlation coefficient of all structures. If the square root of AVE in each structure was larger than the correlation coefficient or latent variables, then it could be stated that, the questionnaire had valid discriminant validity.

### Ethical consideration

The Ethic Committee of KUMS approved the study with the code: IR.KUMS.REC.1397.282. The study objectives were explained to all participants and oral informed consent was obtained from them. The participants were assured about the confidentiality of their personal information and responses.

## Results

Out of 357 distributed questionnaires, 332 were fully completed (92% response rate). Of the 332 students participating in the study, 181 (54.4%) were female and 151 (45.5%) were male. The mean and standard deviation of students’ age was 23.70 ± 2.98 years. Among the age groups, the highest frequency (*n* = 172, 51.8%) belonged to the age group of 25–28 years old. Most of the students were B.Sc. students (*n* = 156.47%) and were mainly from the school of Nursing and Midwifery (*n* = 81. 24.4%). In terms of mobile phone ownership, 81.3% of the students (*n* = 270) had smart phones and 37.3% (*n* = 127) had tablets. Also, 48.8% of the students (*n* = 162) had been using mobile phones for more than 5 years (Table 1). The mean readiness of students for ML was 3.59 ± 0.83 out of 5, which was at moderate level or ready but needs a few improvement.

**Table 1:** Demographic characteristic of respondents

Variables	Items	Number (%)
Sex	Male	151(45.5%)
	Female	181(54.4%)
Age	< 20	5(1.5%)
	20–24	126(38.0%)
	25–28	172(51.8%)
	> 28	29(8.7%)
Educational level	B.Sc.	156(47.0%)
	M.Sc.	118(35.5%)
	Ph.D.	58(17.5%)
School	Medicine	56(16.9%)
	Paramedical	53(16.0%)
	Dentistry	41(12.3%)
	Pharmacy	40(12.0%)
	Nursing and Midwifery	81(24.4%)
	Health	61(18.4%)
Mobile device Ownership		
Smartphone	Yes	270(81.3%)
	No	62(18.7%)
Tablet	Yes	124(37.3%)
	No	208(62.7%)
Years of experience of using mobile devices	< 1	13(3.9%)
	2–3	49(14.8%)
	4–5	108(32.5%)
	> 5	162(48.8%)

The values of Cronbach’s alpha, standardized factor loadings, CR, and AVE are presented in Table 2. Our results indicated that, the value of Cronbach’s alpha for all items in each structure was higher than 0.70 and therefore, they had an acceptable internal consistency. The AVE value results also indicated that, all items had an AVE of higher than 0.50, which was desirable. Furthermore, the CR value for all items was higher than 0.70, which was at the optimal level (Table 2).

**Table 2:** Factor loadings, Cronbach’s alpha, composite reliability and AVE

Constructs	Items	Factors loadings	Cronbach’s Alpha ≥0.7	CR ≥ 0.7	AVE ≥ 0.5
Perceived ease of use	PEU1	0.790	0.730	0.832	0.628
	PEU2	0.710			
	PEU3	0.870			
Perceived usefulness	PU1	0.920	0.835	0.912	0.776
	PU2	0.810			
	PU3	0.910			
Attitude	ATT1	0.839	0.783	0.841	0.592
	ATT2	0.719			
	ATT3	0.748			
Instructor readiness	IR1	0.920	0.821	0.901	0.753
	IR2	0.812			
	IR3	0.869			
Student readiness	SR1	0.850	0.820	0.892	0.735
	SR2	0.910			
	SR3	0.810			
Subjective norm	SN1	0.715	0.836	0.888	0.729
	SN2	0.910			
	SN3	0.921			
Perceived self-efficacy	PSE1	0.959	0.980	0.962	0.895
	PSE2	0.960			
	PSE3	0.920			
Learning autonomy	LA1	0.910	0.925	0.935	0.829
	LA2	0.850			
	LA3	0.969			
Behavioral control	BC1	0.810	0.834	0.833	0.626
	BC2	0.840			
	BC3	0.720			
Intention	INT1	0.789	0.865	0.925	0.806
	INT2	0.920			
	INT3	0.976			

*CR* Composite Reliability, *AVE* Average Variance Extracted

Based on the results of discriminant validity test, the square root of AVE was greater than the correlation coefficient of the structures, so it could be argued that the questionnaire had acceptable discriminant validity (Table 3).

**Table 3:** Square root of AVE and correlation coefficients

Construct	PEU	PU	ATT	IR	SR	SN	PSE	LA	BC	INT
PEU	0.792									
PU	0.776	0.880								
ATT	0.786	0.804	0.769							
IR	0.643	0.633	0.582	0.867						
SR	0.483	0.541	0.555	0.491	0.857					
SN	0.577	0.534	0.623	0.267	0.256	0.853				
PSE	0.499	0.596	0.590	0.652	0.422	0.295	0.946			
LA	0.726	0.781	0.704	0.791	0.680	0.357	0.578	0.910		
BC	0.593	0.699	0.638	0.629	0.419	0.244	0.662	0.609	0.791	
INT	0.747	0.834	0.682	0.658	0.465	0.440	0.585	0.733	0.680	0.897

**Fig. 3:**
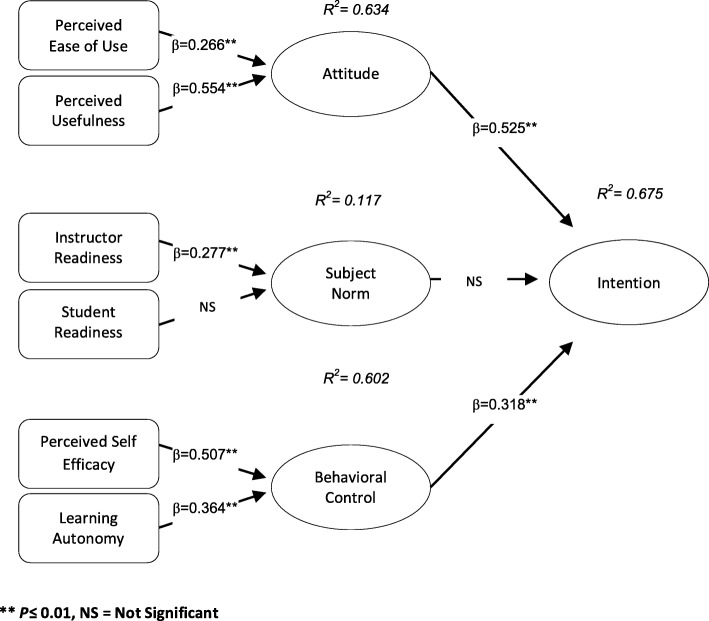
The results of Path coefficients of the research model

In the tested model, path coefficients showed that Attitude (β = 0.525) and Behavioral Control (β = 0.318) had a positive and significant effect on the intention to accept m-learning (*P* ≤ 0.01). But Subject Norm did not have a significant effect on the intention to accept m-learning. The perceived ease of use (β = 0.266) and perceived usefulness (β = 0.554) also had a positive and significant effect on Attitude. The Instructor Readiness (β = 0.277) had a positive and significant effect on Subject Norm, but Student Readiness did not have a significant effect on Subject Norm. The perceived self-efficacy (β = 0.507) and Learning Autonomy (β = 0.364) had a positive and significant effect on Behavioral Control. In general, the Attitude, Subject Norm, and Behavioral Control structures explained the intention to use m-learning (R^2^ = 0.675), (Fig. 3).

## Discussion

In our study, the MLR of students was at moderate level (ready but needs a few improvement). In this regard, the results of a study by Mahat et al. (2012) indicated that Malaysian students have a high level of MLR [28]. In another study in Malaysia showed that the MLR in Malaysian students is at moderate level [29]. In the study of Zayim & Ozel (2015), more than half of nursing students in Turkey were ready for mobile learning [30]. Abu Al-Aish (2014) in a study showed that students in England did not have enough readiness for ML, but had a positive attitude towards it and were willing to use it [31]. One of the critical factors that have a positive correlation with the effectiveness of ML is the learner’s readiness [32]. Therefore, the effectiveness and successful use of mobile learning in the educational process requires the students and instructors of educational institutions and educational centers to have a good understanding of the potentials, limitations and effects of mobile learning [33]. In our opinion, the difference in the level of MLR in students can be related to the amount of access to educational and technological facilities, the acceptance of ML by instructors and students, and the attitude of educational institutions towards ML. Therefore, in order to increase the readiness of students for ML and to use smartphone in the learning process effectively, the aforementioned factors should be considered. The most important finding of present study was that, among the main structures of TPB, The structures of attitude and perceived behavioral control had a positive and significant effect on the intention of students to adopt m-learning. However, subjective norm did not have a significant effect on the intention of students to accept m-learning. Also, the main structures of TPB, including Attitude, Subject Norm, and Behavioral Control were capable of explaining students’ intention to use mobile learning. The findings related to the study hypotheses are discussed below. In our study, the first hypothesis was accepted. The results of hypothesis evaluation showed that, students’ attitude toward m-learning had a positive and significant effect on their intention to adopt m-learning. Therefore, it can be said that positive attitude toward m-learning affects the m-learning acceptance. This finding is consistent with the findings of Seyal et al. [34], Cheon et al. [17], and Reza et al. [22] Studies. According to TPB, attitude is a decisive factor in the use of technology, and expresses the positive or negative feelings of an individual towards a behavior. Learners ‘attitudes affect their intent and learners’ intentions affect their behavior in using m-learning.

The fourth and fifth hypotheses about attitudinal beliefs (including PEU and PU) were also accepted, so that, students’ perception from PEU and PU to m-learning had a positive and significant effect on their attitude toward m-learning. Our findings are in line with the findings of Yeap et al. [20], Renda dos Santos and Okazaki [24], Cheon et al. [17], and Reza et al. [22] Studies. The PU represents the learner’s belief in the usefulness of m-learning and can enhance learner’s satisfaction and performance. PEU refers to the extent to which the learners requires the least mental and physical effort in using the mobile phone [35]. We believe that, the easier psychological and physical use of technology for learners, the more they will be accepted by them. Davis (1993) believes that both PU and PEU play an important role in predicting the attitude of people towards a system, but the effect of PU is 50% more than PEU [36]. Results of several studies indicate that, PU and PEU have a significant effect on the user’s satisfaction and the intention to use a system (ie; m-learning and e-learning), [37, 38]. We also believe that, if students consider the use of mobile useful and easy in learning process, they will have a greater tendency and a more positive attitude toward m-learning.

The second hypothesis in this study which was “the positive and significant impact of students’ subjective norm towards m-learning on their intention to accept m-learning” was not accepted. This finding is consistent with the findings of Seyal et al. study [34], but is not in line with the findings of Cheon et al. [17] and Reza et al. [22] studies.

From the sixth and seventh hypotheses that were related to normative beliefs, only the sixth hypothesis was accepted. The results of sixth hypothesis showed that instructor’s readiness (IR) had a positive and significant effect on subjective norm (SN) in regard to m-learning. This finding is consistent with the findings of Yeap et al. [20], Cheon et al. [17], and Reza et al. [22], studies. The results of seventh hypothesis also showed that SR does not affect SN related to m-learning. This finding is also consistent with the findings of Cheon et al. [17]. Normative beliefs refer to the expectations of others. According to this belief, the expectations of others are a decisive factor in the intention to adopt and accept a technology [18]. Normative beliefs can cover different groups of people, and each can have different views and opinions. The most important reference groups in higher education are faculty members and students [17]. We believe that, the readiness of instructors and students and their view of m-learning are vital for the successful implementation of m-learning system.

The results of third hypothesis showed that BC had a positive and significant effect on the acceptance and intention of using m-learning. The results of 8th and 9th hypotheses about control beliefs also showed that PSE and LA of students had a positive and significant effect on their behavioral control in m-learning. These findings are consistent with the results of Yeap et al. [20]; Renda dos Santos and Okazaki [24]; Chu and Chen [39]; and Reza et al. [22] studies. The BC indicates how much people have control over a behavior. It also indicates the probability of a success of a behavioral attempt [19, 20]. The BC is determined by a set of control beliefs. These beliefs directly or indirectly affect the behavior [20]. In the present study, control beliefs included PSE and LA. The PSE represents a person’s belief in his/her ability to perform a behavior. The LA refers to learners’ control and responsibility over their learning process [20]. We believe that self-efficacy is an important factor in the learning-teaching process, and learners’ self-efficacy in the use of mobile or any other technology plays an important role in their success. Also, in regard to LA, it is believed that individuals with the ability to learn independently are more likely to use mobile learning [40]. Therefore, for the effectiveness of m-learning, self-efficacy of learners and their belief in their abilities should be considered. Additionally, since in mobile learning, the learners must rely on their abilities and potentials, they should have a high independent learning ability to succeed in their mobile learning.

In general, the discovery of human behavior and its dynamics is one of the challenges of behavioral science specialists and researchers. In TPB, behavioral intention is an indicator that shows the actual behavior. Behavioral intention is a combination of structures of attitude, subjective norm and behavioral control. The more positive the attitude, subjective norm, and behavioral control of a person are towards a behavior, the stronger the person’s intention to do that behavior would be [40]. Therefore, in order to prepare the learners for ML, it is necessary to identify the factors that influence the intention of learners in adopting/accepting ML.

### Limitations

This study was confronted with several limitations. The first limitation was the data collection method which was done through self-reporting and that might have affected the accuracy of the results. To overcome this limitation, the researchers tried to reassure the samples about the confidentiality of their information and responses. Another limitation of this study was the differences in the infrastructure of schools/faculties and universities in different regions in accessing technology, which might have affected the generalizability of the results.

## Conclusions

The results showed that MLR of students was at moderate level. ATT and BC had a significant effect on m-learning acceptance. But, SN had no significant effect on m-learning acceptance. The main structures of TPB, including ATT, SN and BC were able to explain the intention of students to accept m-learning. Therefore, the TPB-based model in this study was useful for identifying the psychological-behavioral factors affecting m-learning acceptance. Mobile learning provides a good opportunity for lifelong learning of students. In order to evaluate and identify the behavioral and psychological factors that affect m-learning adoption/acceptance, it is suggested to use other technology adoption models and theories (Technology Acceptance Model and Unified Theory of Acceptance and Use of Technology, Diffusion of Innovations theory). Qualitative studies can also be useful in identifying the pedagogical, technological and cultural-social challenges in the design and implementation of mobile learning system. Content is an important element of learning. Therefore, identifying the principles and standards of content development for mobile learning in the form of qualitative studies, is suggested by investigating the views of experts in the fields of educational technology, e-learning, and curriculum planning.

## Data Availability

Data available by contacting the corresponding author.

## Abbreviations

ATT: Attitude
CVI: Content validity index
CVR: Content validity ratio
INT: Intention
IR: Instructor readiness
KUMS: Kermanshah University of Medical Sciences
LA: Learning autonomy
M-Learning: mobile learning
MLR: mobile learning readiness
PBC: Perceived behavioral control
PEU: Perceived ease of use
PSE: Perceived self-efficacy
PU: Perceived usefulness
SN: Subjective norm
SR: Student readiness
TPB: theory of planned behavior
